# Multiple-Criteria Decision Analysis for Assessments of Chemical Alternatives (MCDA-ACA)

**DOI:** 10.1021/acs.est.4c03980

**Published:** 2024-10-18

**Authors:** Rachel L. London, Juliane Glüge, Martin Scheringer

**Affiliations:** Institute of Biogeochemistry and Pollutant Dynamics, ETH Zürich, 8092 Zürich, Switzerland

**Keywords:** multiple-criteria decision analysis, assessment of alternatives, regrettable substitution, substance of very high concern (SVHC), minimum aggregation, chemical hazard assessment

## Abstract

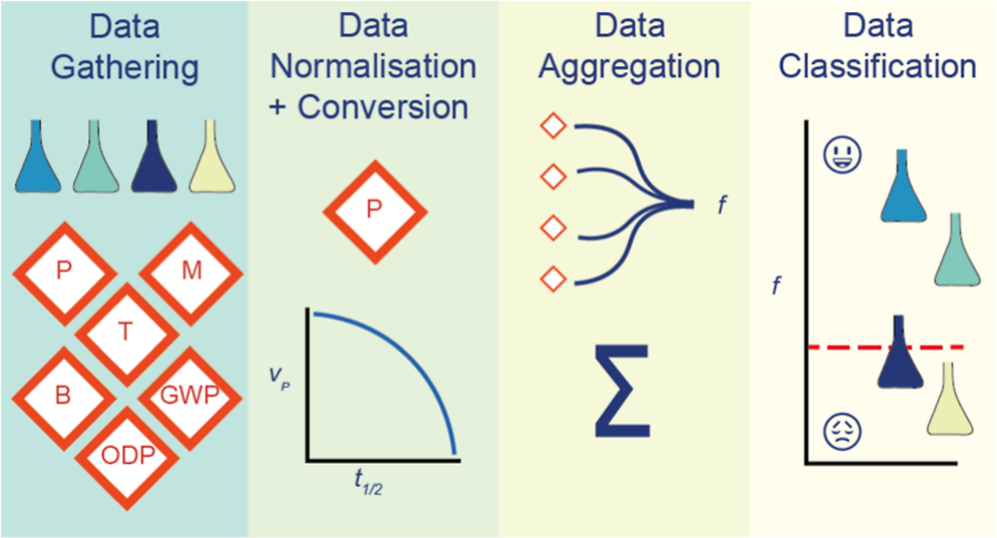

A comprehensive assessment of chemical alternatives (ACA) is necessary to avoid regrettable substitution. In a preceding study, an analysis of six hazard assessment methods found that none of them are fully aligned with the hazard assessment criteria of Article 57 of the European REACH regulation, indicating a need for a method better reflecting hazard assessment schemes in European chemical regulations. This paper presents a multiple-criteria decision analysis (MCDA) method for the ACA that takes the criteria of Article 57 of REACH into account. Investigated and presented are objective hierarchies, the aggregation of objectives, the curvature of the value functions, weights, and the introduction of a classification threshold. The MCDA-ACA method allows for the aggregation of hazards in such a way that poor performance in one hazard cannot be compensated for by good performance in another hazard. The method parameters were developed and tested using two data sets with the aim to classify chemical alternatives into acceptable (nonregrettable) and unacceptable (regrettable) alternatives according to the regulations set in Europe. The flexibility of the general method was explored by adapting the method to align with two hazard assessment schemes, Article 57 of REACH and GreenScreen. The results show that MCDA-ACA is so flexible and transparent that it can easily be adapted to various hazard assessment schemes.

## Abstract 2

We propose parameters for a multiple-criteria decision analysis method to comprehensively assess chemical alternatives and present a practical policy tool necessary to prevent regrettable substitutions.

## Introduction

Substituting hazardous chemicals is often challenging because the original chemicals have, in most cases, very specific properties and use areas. In general, three different approaches are available for chemical substitution. One option is a chemical-by-chemical replacement, which is also often called “drop-in chemical replacement”.^1^ A second option is to find an alternative way of achieving the function of the chemical in the product, for example, by redesigning the product or by choosing a different material. Third, a chemical can also be substituted by a change of the system so that the function of the chemical is not required anymore.^1^ However, the second and third options are often more complex and may involve higher investment costs. For this reason, a chemical-by-chemical replacement has often been preferred by companies over the other options.^2^ Unfortunately, this has also led to regrettable substitutions, where one hazardous chemical has been replaced by another similar one. To avoid the obstacles of regrettable substitution and “lock-in” of hazardous chemicals, potential chemical alternatives should be comprehensively assessed for their hazards before they are introduced into the market.

Jacobs et al.^3^ listed several assessment-of-alternative (AoA) frameworks and identified six common components: hazard assessment, technical feasibility assessment, economic assessment, exposure characterization, life-cycle assessment/life-cycle thinking, and decision making. Tickner et al.^4^ identified literature gaps and a research agenda to advance the AoA field in the six components listed by Jacobs et al.^3^ One of the research needs they identified was to develop decision-making support methods and tools for use in private and regulatory contexts and, specifically, to adapt emerging and existing decision-making support tools for the weighting (and aggregation) of different hazard data. A recent article on this topic (Bechu et al.^5^) concluded that there has been progress in the method and tool development in decision-making. However, it was also stated that further guidance on the use of formal decision-making support tools such as multiple-criteria decision analysis (MCDA) for alternative assessment is needed.^5,6^

In our preceding paper,^7^ we analyzed whether several existing methods for hazard assessment and their decision-making concepts are in line with the hazard criteria of Article 57 of the EU regulation on the Registration, Evaluation, Authorisation, and Restriction of Chemicals (REACH). The criteria in Article 57 describe substances that are of very high concern (SVHCs) and may be included in Annex XIV of REACH, which is the list of substances subject to authorization. In London et al.,^7^ we showed that none of the investigated hazard assessment methods use the same criteria as described in Article 57 of REACH. The same conclusion holds true for the hazard assessment methods that have used MCDA in the past. MCDA is a decision-making support tool that can help decision-makers to choose rationally between multiple options when there are several conflicting objectives.^8,9^ In London et al.,^7^ we concluded that it might be possible to align a method based on MCDA with the criteria of Article 57 on REACH if a more sophisticated objective hierarchy is applied to the hazard criteria.

Several applications of MCDA to the hazard assessments of chemical alternatives have been presented recently.^10−14^ However, these studies did not investigate the variability of the results obtained with different MCDA method parameters, such as different types of aggregations or different value functions. Typically, equally weighted objectives were assessed in combination with linear value functions and aggregated by taking the arithmetic mean. External regulatory thresholds for hazards, such as a degradation half-life of 180 days for persistent chemicals, were not used, and the methods did not reflect the combined hazard criteria used in Article 57 of REACH (e.g., very persistent (vP) and very bioaccumulative (vB)).

Here, we propose an MCDA method based on multi-attribute value theory (MAVT) with discrete value functions^15^ for the assessment of chemical alternatives (ACA), the MCDA-ACA method. MCDA-ACA takes the hazard criteria of Article 57 of REACH into account but also allows for the integration of various additional objectives, including global warming potential (GWP) and ozone depletion potential (ODP). MCDA-ACA combines the objectives in such a way that poor performance in one objective cannot be compensated for by good performance in another objective. Although the method was initially developed to be applied in the context of European regulation, it is flexible enough to be applied to other jurisdictions as well. In this paper, the MCDA-ACA method parameters are investigated and set by using a hypothetical substance data set and subsequently tested using a previously published data set of real substance data. It is also shown that the objective hierarchy can be adapted to other objectives, such as obtaining the same output as GreenScreen or mimicking exactly the criteria laid down in Article 57 of REACH. Mimicking GreenScreen is interesting as GreenScreen is a decision tree that is recommended and used in various jurisdictions including in the US^16^ and the EU.^17^ Recreating GreenScreen also makes it possible to understand whether GreenScreen has a consistent decision logic through all its hazard classes (called “Benchmarks”). We also provide a Supporting Information file (Supporting Information-3) where the newly developed methods can be used by practitioners in the future.

## Methods

### Criteria for the Development of the MCDA-ACA Method

The idea of the MCDA-ACA method is to classify chemical alternatives into acceptable (nonregrettable) and unacceptable (regrettable) substances. This is done by taking the current European chemical legislation, REACH, and objectives recommended in other legislations into account. The objectives selected include, with one exception (the physical hazard of flammability), all minimum hazard criteria that were defined by the Organisation for Economic Co-operation and Development (OECD) in 2021 and additionally some of the so-called “moving beyond the minimum” criteria of the OECD.^18^ One aim is also that the new method can rank the nonregrettable substances according to their hazard. The objectives included are

- PBT_eco_ and PB, to avoid persistent (P), bioaccumulative (B), and ecotoxic (T_eco_) as well as vP and vB substances, in line with the criteria of Article 57 of REACH.
- human toxicity (T_hu_), in order to avoid carcinogenic and mutagenic substances and those that are toxic to reproduction or have other effects on humans such as endocrine disrupting chemicals, in line with the criteria of Article 57 of REACH
- PMT_eco_ and PM, to avoid persistent, mobile (M), and ecotoxic as well as vP and very mobile (vM) substances, in line with the current CLP regulation
- BT_eco_, to avoid vB and very ecotoxic (vT_eco_) substances, in line with the criteria of GreenScreen for substances that are GreenScreen Benchmark 1 (highest hazard level)
- PT_eco_, to avoid vP and vT_eco_ substances, in line with the criteria of GreenScreen for substances that are GreenScreen Benchmark 1 (highest hazard level)
- GWP, in order to avoid substances that are potent greenhouse gases
- ODP, to avoid substances that can destroy the ozone layer

**Figure 1:**
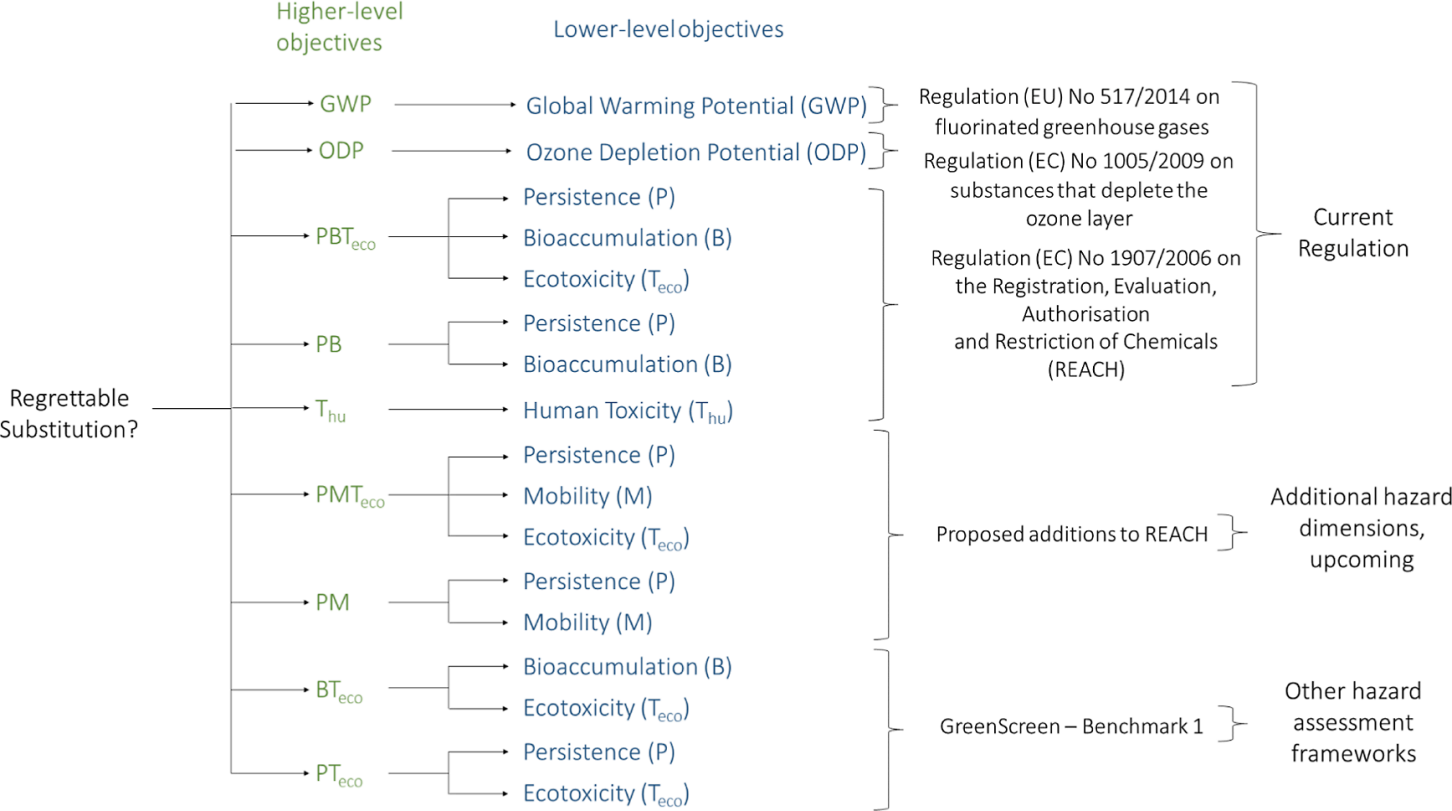
Objective hierarchy of the MCDA-ACA method. GWP and ODP are higher-level objectives that consist of three letters, whereas *P*, *B*, *M*, *T*_eco_, and *T*_hu_ are all individual lower-level objectives that are aggregated into various higher-level objectives.

Figure 1 shows the objective hierarchy of the MCDA-ACA method, showing how the objectives relate to one another and the reasons for their inclusion. The thresholds that define when a lower-level objective is actually very high, high, moderate, or low are provided in Supporting Information-1, Section S2. The thresholds are based–if available–on the thresholds given in Article 57 of REACH. If no thresholds are available in Article 57, they are based on other relevant European chemical legislation.

According to the analysis by London et al.,^7^ a method based on MCDA was deemed useful due to its flexibility and the possibility to explicitly set parameters. The five MCDA parameters that can be varied and are investigated here are the objectives of the assessment (*i*), the attributes by which these objectives are measured, the value function that is used to convert these attributes into compatible values (ν_*i*_) for each objective *i*, the aggregation equation used to combine these values, and, when required, the weights assigned to each objective (*w*_*i*_). The next section explains these terms in more detail.

### Introduction to MCDA, Terminology, and Method Parameters

MCDA is a method that assists decision makers in identifying the best alternative in a given context for a given set of objectives. The objectives are the criteria according to which the alternatives are evaluated. The objectives for any given MCDA should be complete, independent, measurable, concise, without redundancy,^9^ and ideally fewer than 15 in number.^19^ Exceptions to this rule are possible if minimum or maximum aggregation is used (see below), as this can resolve the redundancy and/or reduce the number of criteria. The relationship among objectives is visually defined in terms of an objective hierarchy (e.g., the lower-level objectives of persistence, bioaccumulation, and ecotoxicity contributing to the higher-level objective of PBT). Objectives are measured by attributes (e.g., degradation half-life of a chemical in water). All attributes are converted into comparable values using a value function that should have a curvature that reflects the specific preferences of the decision maker. These values are aggregated according to the objective hierarchy and by means of an aggregation logic that reflects the preference of the decision maker. Some aggregation equations necessitate the use of weights, values that quantify the relative importance of objectives.^9^ The above terms are referred to in this article as “method parameters”. These method parameters make MCDA a highly flexible method, and they should be adjusted to the context of each particular MCDA, including the preferences of the decision maker. Method parameters should also be justified, which makes MCDA a transparent decision-making method. For further definition and explanation of the terms introduced in this section, see Supporting Information-1 Section S1.

### Selection of MCDA Method

This paper proposes an MCDA method to assess chemical alternatives using the MCDA method “multi-attribute value theory” (MAVT) with discrete value functions. There are different types of MCDA methods. To evaluate chemical alternatives within a regulatory context, the MCDA method chosen has to be robust (meaning that the result for a given alternative must be independent of the other alternatives included in the analysis). This excludes methods such as AHP, ELECTRE, PROMETHEE, and possibly DSA.^20^ MAVT and “multi-attribute utility theory” (MAUT) are both robust methods that can be used when a problem has multiple objectives, with MAVT being a simplified form of MAUT.^21^ When the values of attributes are certain, a value function can be used, and so MAVT is appropriate. When uncertainty or the risk tolerance of a decision maker needs to be included, a utility function can be used, and MAUT is appropriate.^15^ In this work, it was found that discrete value functions were required to make the method align with the regulation. Therefore, MAVT rather than MAUT was used in this paper.

### Data Sets

The MCDA-ACA method parameters were investigated and set by using a hypothetical substance data set and subsequently tested by means of a previously published set of real substance data. The hypothetical substance data set is derived from the hazards, and hazard levels, that characterize the identified objectives. The investigated lower-level objectives include P, B, and T_eco_. The assessment for mobility is the same as for bioaccumulation, and it was possible to set the value function, the aggregation, and the scaling factor for PMT_eco_ in the same way as for PBT_eco_. Value function, aggregation, and scaling factor for vPvM were set in the same way as for vPvB. Mobility (M) was therefore not added as a hazard to the hypothetical substances. Human toxicity (T_hu_) was added as a hazard to the hypothetical substances to see if a higher-level objective with just one lower-level objective could also be included in the same system. Therefore, the hypothetical substance data set contains 256 combinations (4^4^) of the four hazards (P, B, T_hu_, and T_eco_) and four qualitative hazard levels (very high, high, moderate, and low). Additionally, for all hypothetical substances, the different hazard combinations were labeled manually as if they were classified according to the regulations shown in Figure 1. As can be seen in Supporting Information-2, 148 of the 256 substances have SVHC characteristics.

The real substance data set was derived from an MCDA study by Zheng et al.,^11^ who investigated 16 alternative substances to the flame retardant, decaBDE. There are 20 quantitative attributes in the real substance data set, including five for persistence, one for bioaccumulation, one for mobility, and 13 for toxicity. Some data points were determined experimentally, and others were based on quantitative structure–activity relationships (QSARs). For our assessment, we used the raw data from Zheng et al.^11^ in the data gathering step. However, the data normalization was not adopted from Zheng et al.,^11^ but carried out by us using the thresholds from the Guidance on Information Requirements and Chemical Safety Assessment Chapter R.11: PBT/vPvB assessment.^22^ The normalized data were then used to test the data aggregation and data classification. The data sets lacked information on uncertainty; however, this was considered acceptable as the focus of our study was to develop and test a new method.

### Steps to Use the MCDA-ACA Method

**Figure 2:**
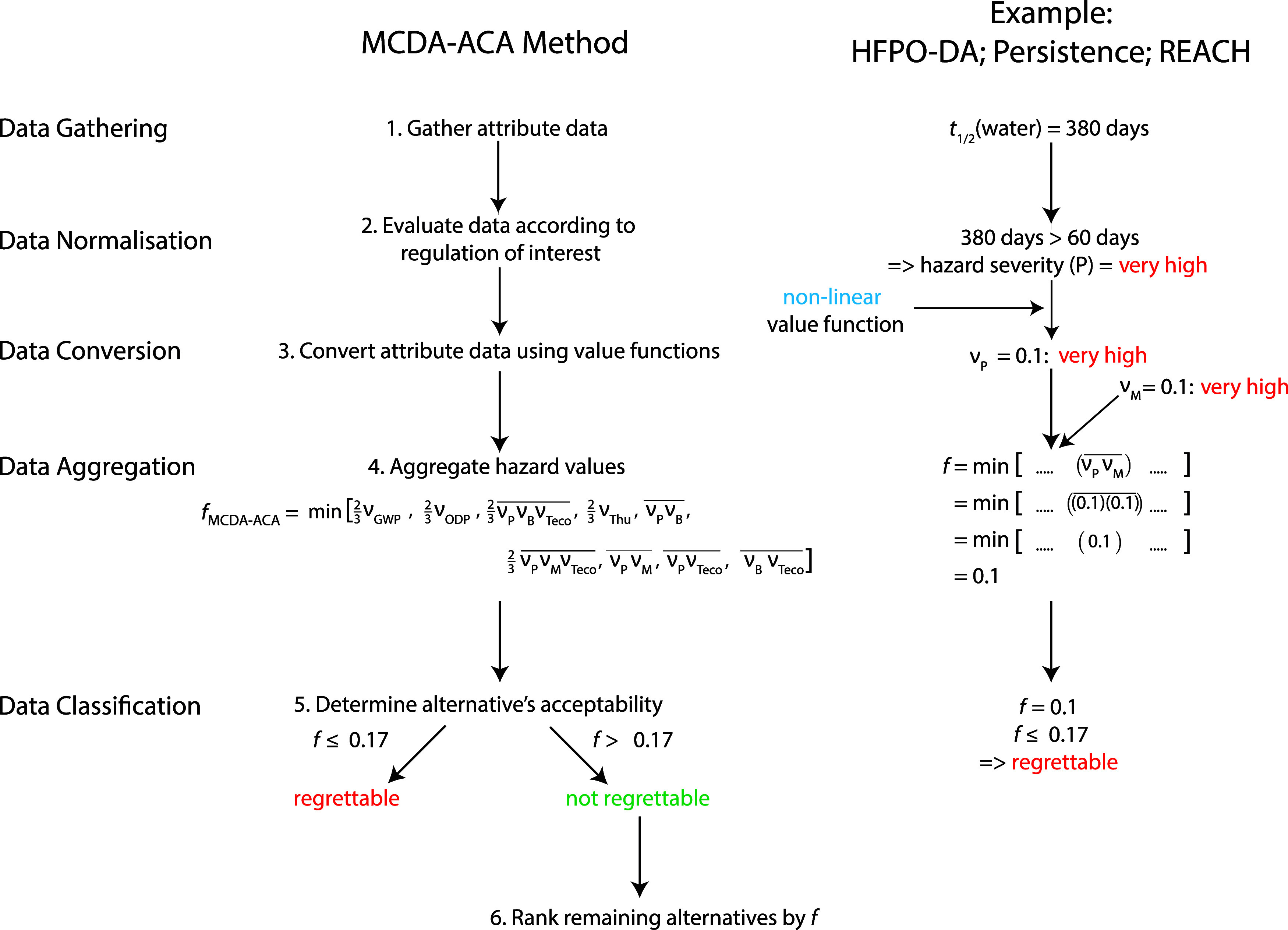
Flowchart showing the MCDA-ACA method. The flowchart on the left shows the general steps, and the flowchart on the right shows the steps for the persistence assessment of HFPO-DA. The notation  stands for the arithmetic mean of *X* and *Y*.

The MCDA-ACA method involves five steps through which a chemical can be classified as acceptable or unacceptable. These steps are data gathering, data normalization, data conversion, data aggregation, and data classification. The following paragraphs describe these five steps using the persistence assessment of 2,3,3,3-tetrafluoro-2-(1,1,2,2,3,3,3-heptafluoropropoxy propanoic acid (HFPO-DA) as an example. Figure 2 shows the corresponding flowchart that includes on the left a general description of the five steps and on the right the steps in the persistence assessment of HFPO-DA.

### Data Gathering

Attribute data need to be collected for the lower-level objectives of GWP, ODP, P, B, T_eco_, T_hu_, and M. In the example, the attribute “half-life in fresh water” of HFPO-DA was selected as 380 days^23^ (converted from BIOWIN 3 using the equation published in Scheringer et al.^36^) and was used for the persistence assessment.

### Data Normalization

To ensure alignment of the assessment with relevant regulations, external thresholds are used to categorize the hazard levels of the attributes. Each attribute is categorized into one of the four hazard levels, “very high”, “high”, “moderate”, or “low”. The individual thresholds that we recommend for MCDA-ACA are given in Supporting Information-1 Section S2. For the use of the MCDA-ACA method in the context of European regulation, the thresholds from Annex XIII of REACH^24^ are recommended. A category of “high” is given to attributes with half-lives in fresh water above 40 days, while a “very high” is given to those with half-lives in fresh water above 60 days. Therefore, with a half-life in fresh water of 380 days, HFPO-DA would receive the categorization “very high” for this attribute. If the method is intended to be used in other regulations or regions, then the thresholds should be adapted accordingly.

### Data Conversion

Once a hazard level has been assigned to all attributes related to a given objective (*i*), the hazard levels are converted to a value, ν_*i*_, where 0.0 ≤ ν_*i*_ ≤ 1.0, which follows a nonlinear (convex) value function. The values are 0.1 for “very high”, 0.25 for “high”, 0.6 for “moderate”, and 1.0 for “low”. Values closest to zero represent the least desirable outcome (highest hazard), while values closest to one represent the most desirable outcome (lowest hazard). In the example in Figure 2, for HFPO-DA, the attribute of half-life in fresh water would be “very high” according to the threshold in REACH, if this is the only attribute considered for the objective of “persistence”, then ν_P_ = 0.1. If several attributes are available for one objective, then it is proposed to use a minimum aggregation, meaning that the worst hazard level of all attributes is selected for the objective.

### Data Aggregation

The MCDA-ACA method is a mixed aggregation model that uses both additive and minimum aggregation. Additive aggregation is used for the aggregation of lower-level objectives into higher-level objectives. For example, the higher-level objective of (low) PM can be broken down into the lower-level objectives of (low) persistence and (low) mobility, where persistence and mobility are given equal weights. In our example, an additive aggregation (i.e., a weighted arithmetic mean, where each lower-level objective has *w*_*i*_ = 0.5) is taken of persistence (ν_P_ = 0.1) and mobility (ν_M_ = 0.1), resulting in ν_PM_ = 0.1.

Minimum aggregation is then used to aggregate the higher-level objectives into an MCDA output for the given alternative, denoted by *f*. This means that the lowest hazard score of all higher-level objectives is selected as the MCDA output, *f*, for a given alternative. For HFPO-DA, this results in a *f*_HFPO-DA_ of 0.1 (“not recommended for use as an alternative”). The use of minimum aggregation to combine the higher-level objectives prevents poor performance in one objective from being compensated for by good performance in another objective. Minimum aggregation also ensures that the redundancy in the lower-level objectives does not bias the MCDA output, *f*.

### Data Classification

The MCDA output, *f*, is then used to classify the alternatives in terms of their chemical acceptability. This is performed in two ways. First, alternatives with an *f* below the classification threshold of 0.17 are classified as “regrettable”, while alternatives with an *f* above the classification threshold of 0.17 are classified as “not regrettable”. The derivation of the classification threshold of 0.17 is explained below in the subsection: Parameters of the MCDA-ACA Method.

Second, once the regrettable alternatives have been removed, the remaining alternatives in the assessment can be ranked in terms of their relative chemical acceptability (i.e., which alternative has an *f* value closest to 1.0).

Should numerous alternatives be classified as “not regrettable”, additional hazards can also be evaluated to differentiate between them. This includes expanding the objective hierarchy to include new objectives (e.g., physical hazards such as flammability, ecotoxicity alone, and persistence alone) as well as reevaluating current objectives with lower thresholds (e.g., lowering the threshold for GWP).

In the example, *f*_HFPO-DA_ = 0.1 and thus is lower than 0.17. HFPO-DA is therefore classified as “regrettable” and would not be recommended to be used as an alternative.

### Parameters of the MCDA-ACA Method

In eq 1, the objective hierarchy shown in Figure 1 and used in the data aggregation step shown in Figure 2 is formalized: first, the arithmetic means of the ν_*i*_ values of the lower-level objectives are taken (denoted by, e.g., ), and then the lowest hazard score of all higher-level objectives is selected (command “min”) as the MCDA-ACA output of the alternative considered
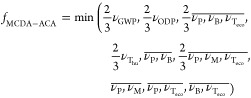
1

The scaling factor of 2/3 is explained below. To ensure that the MCDA-ACA method correctly reflects the regulation and guidance referred to in Figure 1, the method parameters need to be optimized, including the curvature of the value function (for the data conversion step), the inclusion of a scaling factor, here 2/3 (for the data aggregation step), and the classification threshold (for the data classification step).

The parameters were optimized by comparing the known labels of “regrettable” vs “not regrettable” for the set of 256 hypothetical chemicals with the MCDA-ACA output for the same chemicals and refining the MCDA-ACA parameters until agreement between the labels and the MCDA-ACA output was reached. An important consideration here was that the hazard combination of high P, high B, and high T_eco_ should have—according to Article 57 of REACH—the outcome “regrettable”, while at the same time, very high P, high B, and moderate T_eco_ should be “not regrettable”. This is only possible if a nonlinear (convex) value function is used. The reason is that, with a convex value function, the differences between the values increase from very high to high to moderate and low. The average of very high, high, and moderate thus has a higher score with the convex value function (the score would be 0.317 if 0.1, 0.25, and 0.6 are used for very high, high, and moderate, respectively) than the average of high, high, and high (score 0.25). With a linear value function, the score in both examples would be 0.25. A convex value function was therefore chosen for MCDA-ACA. Finding suitable discrete values for the convex value function was performed manually by using Table 1 and is described in detail in the Supporting Information-1 Section S3.1. The final hazard values are 0.1, 0.25, 0.6, and 1 for very high, high, moderate, and low, respectively.

**Table 1:** Higher-Level Objectives of the MCDA-ACA Method, with Examples of Regrettable and Nonregrettable Hazard Combinations, Together with Their Corresponding Score^a^

	regrettable		not regrettable		
higher-level	hazard	score	hazard	score	factor 2/3
objectives	combinations		combinations		
PBT_eco_	hP, hB, vT_eco_	0.13	vP, vB, mT_eco_	0.178^b^	x
	hP, hB, hT_eco_	0.167	vP, hB, mT_eco_	0.21	x
			hP, hB, mT_eco_	0.24	x
T_hu_	vT_hu_	0.07	mT_hu_	0.4	x
	hT_hu_	0.167			x
PB	vPvB	0.1	vP, hB	0.175	
			vB, hP	0.175	
			hP, hB	0.25	
PT_eco_	vP, vT_eco_	0.1	vP, hT_eco_	0.175	
			vT_eco_, hP	0.175	
			hP, hT_eco_	0.25	
BT_eco_	vB, vT_eco_	0.1	vB, hT_eco_	0.175	
			vT_eco_, hB	0.175	
			hB, hT_eco_	0.25	
PMT_eco_	hP, hM, vT_eco_	0.13	vP, vM, mT_eco_	0.178^b^	x
	hP, hM, hT_eco_	0.167	vP, hM, mT_eco_	0.21	x
			hP, hM, mT_eco_	0.24	x
PM	vPvM	0.1	vP, hM	0.175	
			vM, hP	0.175	
			hP, hM	0.25	
GWP	vGWP	0.07	mGWP	0.4	x
	hGWP	0.167			x
ODP	vODP	0.07	mODP	0.4	x
	hODP	0.167			x

a The value function is non-liner and convex (very high (v) = 0.1, high (h) = 0.25, moderate (m) = 0.6, and low (l) = 1.0). “*x*” indicates high-level objectives where the factor of 2/3 was included.

b This combination of lower-level objectives is covered by another higher-level objective.

The derivation of the classification threshold and the scaling factor of 2/3 for some of the higher-level objectives can be understood by looking at Table 1. All hazard combinations that are listed under “regrettable” in Table 1 need to have a score that is lower than the score for the hazard combinations that are listed under “not regrettable.” Which hazard combinations fall under “regrettable” and which under “not regrettable” is defined by Article 57 of REACH and the other European Regulations, which are given in Figure 1.

When looking at the hazard combinations that have two lower-level objectives (PB, PT_eco_, BT_eco_, PM), one can see that the lowest score for the “not regrettable” hazard combinations is 0.175. This means that all scores for the “regrettable” hazard combinations need to be lower than 0.175. However, hazard combinations such as high P, high B, and high T_eco_ or high T_hu_ that describe regrettable substitutes according to Article 57 of REACH would receive a score of 0.25 as the average hazard level is “high.” The scaling factor lowers this score to 0.167 and is, therefore, needed to ensure the correct classification of the hazard combinations. For more information, see Supporting Information-1 Section 3.2.

The highest score for the regrettable hazard combinations obtained is therefore 0.167, and the lowest score for the nonregrettable hazard combinations is 0.175. A classification threshold of 0.170 can therefore separate regrettable and nonregrettable hazard combinations. Importantly, the scaling factor should not be confused with a weighting factor that is sometimes needed in other MCDA methods. The use of minimum aggregation for the higher-level objectives does not require any weighting factors here.

### Creating MimicREACH and MimicGreenScreen

In order to show that the MCDA-ACA method can be tailored to other decision logics and objective hierarchies, two additional MCDA methods were created: MimicREACH and MimicGreenScreen. The aim of MimicREACH is to classify chemical alternatives into substances that meet the criteria for SVHCs as defined in Article 57 of REACH and those that do not meet the criteria. MimicGreenScreen intends to classify chemical alternatives according to the four Benchmark categories that are defined in Annex 3 of GreenScreen.^25^

As a first step, objective hierarchies were created using the lower-level objectives identified in Article 57 of REACH^24^ and in Annex 3 of GreenScreen.^25^ Second, an aggregation equation was created for both models. Finally, the data classification step was carried out to replicate the original methods.

## Results

### MCDA-ACA

With the objective hierarchy given in Figure 1, it was possible to find suitable MCDA method parameters (see Supporting Information-2 “MCDA-ACA method—Hyp. Subs”). Of the hypothetical substances, 61% were classified as “regrettable” (*f* ≤ 0.170), while 39% were classified as “not regrettable” (*f* > 0.170). All substances that would be classified as SVHC under REACH were also classified as “regrettable” by MCDA-ACA. For 3% of the substances, MCDA-ACA considered their hazard combinations “regrettable,” while they would not be classified as SVHC under REACH. Specifically, this occurred for substances with the hazard combinations vPvT_eco_ and vBvT_eco_. Within the group of hypothetical substances classified as “not regrettable,” certain substances still have a single “very high” hazard. This occurs where a hazard is not included individually as a higher-level objective but combined (by taking the arithmetic mean) with other hazards in the formation of higher-level objectives; see Figure 1. For example, Substance 64 in Supporting Information-2 (vP, lower-level, *f* = 0.47) is classified as “not regrettable,” while Substance 253 in Supporting Information-2 (vT_hu_, higher-level, *f* = 0.07) is classified as “regrettable.” For all results, see Supporting Information-2 “MCDA-ACA method—Hyp. Subs”. Important to note here is that the assessment was conducted only with the lower-level objectives P, B, T_eco_, and T_hu_. If the substances represented real world examples, those that were deemed nonregrettable would also need to be evaluated for M as well as GWP and ODP to confirm that they are really nonregrettable. When the MCDA-ACA method was applied to the 17 real substances, all substances were classified as “regrettable” (*f* ≤ 0.170). However, there is some small variation in *f* among the alternatives (0.07 ≤ *f* ≤ 0.10), indicating that some alternatives are worse than others. For all results, see Supporting Information-2 “MCDA-ACA method—Real Subs.”

Practitioners who would like to use the MCDA-ACA method on their own can use the “MCDA-ACA method - forUsers” in the Supporting Information-3. The structure of the sheet is similar to that in Supporting Information-2, except that users can enter their own data (hazard severities) here and that there are further instructions as to where which values must be entered.

### MimicREACH

**Figure 3:**
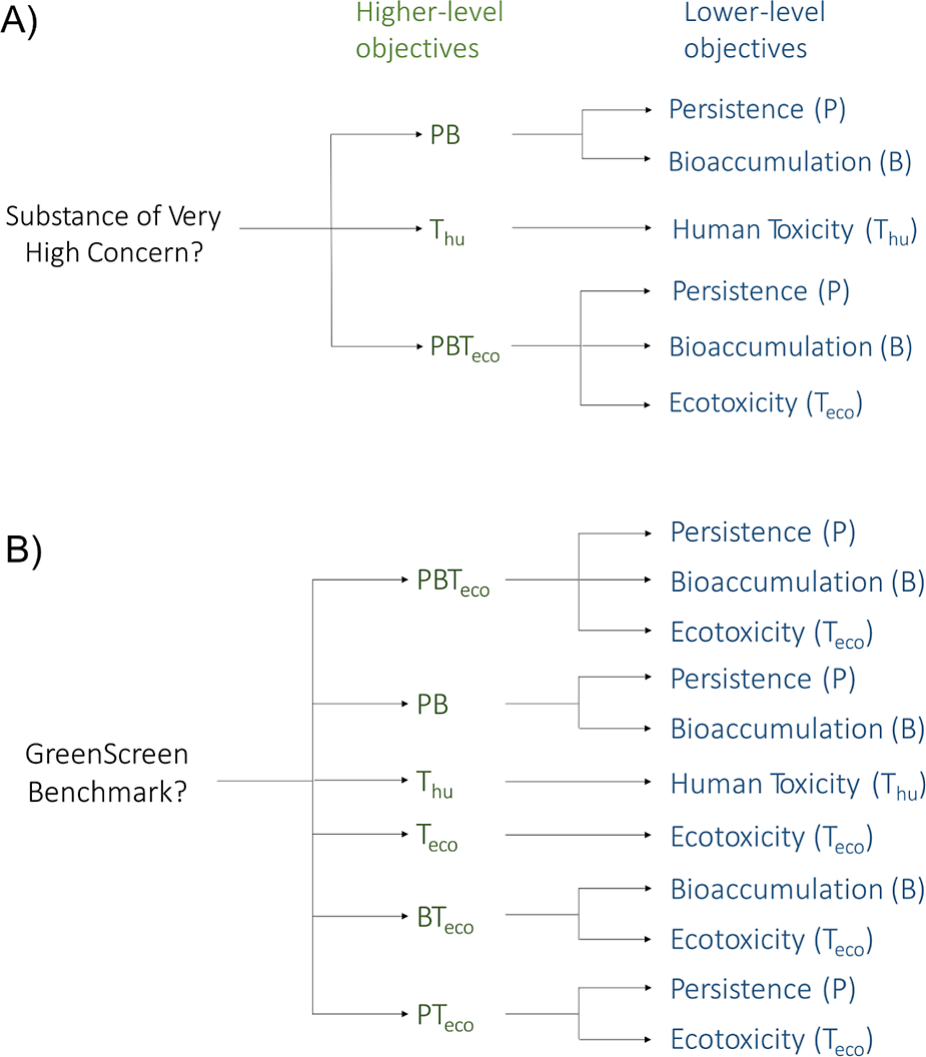
Objective hierarchy of (A) MimicREACH and (B) MimicGreenScreen.

A small number of adjustments to the MCDA-ACA method made it possible to replicate the decision logic of the hazard assessment according to Article 57 of REACH, see also Supporting Information-2 “MCDA—MimicRACH”. The objective hierarchy is shown in Figure 3A). The main change is that fewer higher-level objectives were included in the objective hierarchy than those for MCDA-ACA. The corresponding aggregation equation is given in eq 2.
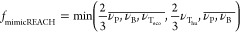
2

The value functions and the classification threshold for SVHC vs not SVHC are the same as in MCDA-ACA. When applied to the 256 hypothetical substances, MimicREACH correctly identifies all 148 SVHCs. An implementation of MimicREACH in MS Excel is provided in the Supporting Information-3 under ´́MCDA - mimicREACH - forUsers”.

### MimicGreenScreen

With MimicGreenScreen, we intend to replicate the decision logic of GreenScreen. The objective hierarchy is shown in Figure 3B. The main difference from MCDA-ACA is that PMT_eco_, PM, GWP, and ODP are not included as higher-level objectives in MimicGreenScreen, while T_eco_ is added additionally. The parameters include again a mixed aggregation hierarchy (additive and minimum), nonlinear (convex) value functions, and equal weighting for the lower-level objectives. Because there are four so-called Benchmarks in GreenScreen, three classification thresholds are needed instead of one in MCDA-ACA. The thresholds were set at 0.170, 0.41, and 0.65 (the derivation of the thresholds is shown in the Supporting Information-1 Section S4), and the aggregation equation is shown in eq 3.
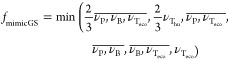
3

With these parameter combinations and thresholds, 89% of the hypothetical substances were classified consistently with GreenScreen. This is an important finding that shows that the decision logic of GreenScreen, in large part, has a consistent structure that can be replicated with an MCDA method. At some points, however, GreenScreen contains ad-hoc weightings of certain hazards that cannot be replicated by the value functions underlying MimicGreenScreen and, therefore, lead to different results for MimicGreenScreen and GreenScreen. Some of these cases are listed in Table 2. These cases are characterized as follows:

- Substances with PBT or even vPBT properties, but low human toxicity are only Benchmark 2 in GreenScreen, but Benchmark 1 in the MCDA decision logic; here, GreenScreen requires very high T_eco_ in addition to (v)PB for the substances to be Benchmark 1 (6 substances, rows 1 and 2 in Table 2)
- Substances with very high ecotoxicity, both alone and in combination with other hazards, are only Benchmark 2 in GreenScreen, but Benchmark 1 in the MCDA decision logic (16 substances, rows 3 and 4 in Table 2)
- Substances with high ecotoxicity, low human toxicity, and low or moderate P and B are only Benchmark 3 in GreenScreen, but Benchmark 2 in the MCDA decision logic (3 substances, row 5 in Table 2)
- Four specific cases where GreenScreen and the MCDA decision logic assign Benchmarks 2 instead of 3 and vice versa (rows 6 to 9 in Table 2)

**Table 2:**
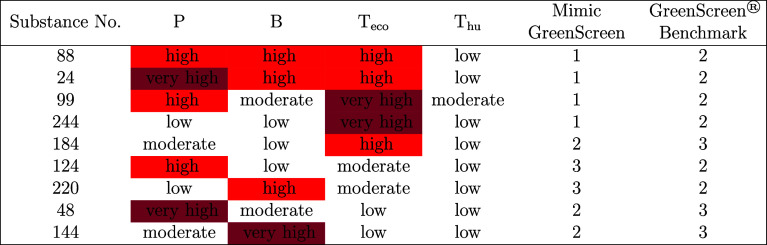
Selected Examples of MimicGreenScreen and GreenScreen Benchmarks from the Hypothetical Data Set, Where 1 = “Avoid—Chemical of High Concern,” 2 = “Use but Search for Safer Substitutes,” 3 = “Use but Still Opportunity for Improvement,” and 4 = “Prefer—Safer Chemical”

Regarding Benchmarks 2 to 4 of GreenScreen, there is no clear “right” or “wrong” as there is no legal reference point for these substances. However, substances that are Benchmark 1 in MimicGreenScreen, such as PBT substances, would potentially be classified as SVHCs under REACH, and we therefore think that Benchmark 1 (Avoid—chemical of high concern) is more appropriate in this case than Benchmark 2 (Use, but search for safer substitutes).

### Effect of Different Objective Hierarchies

**Figure 4:**
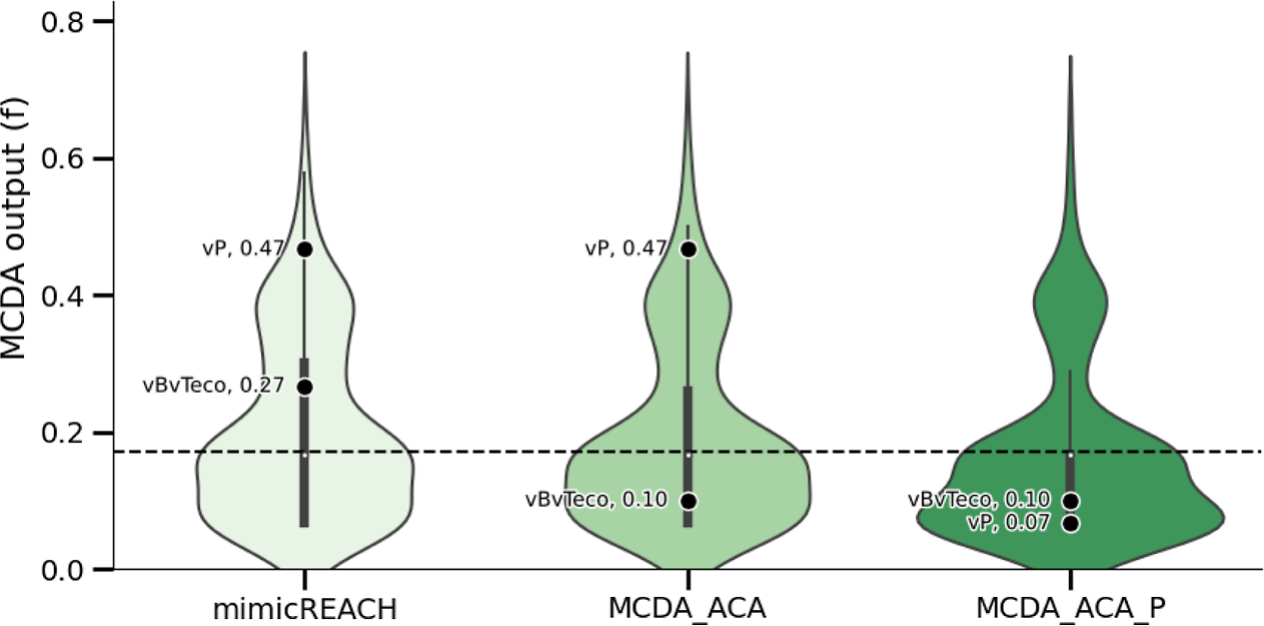
Distribution of MCDA output (*f*) for the different objective hierarchies of MimicREACH, MCDA-ACA, and MCDA-ACA + P. The distribution of *f* was calculated for the hypothetical substance data set. Two hazard combinations are highlighted: vBvT_eco_ (very bioaccumulative and very ecotoxic) and vP (very persistent, low hazards in the other end points). The dashed line denotes the classification threshold of *f* = 0.17, below which a hazard combination is classified as “regrettable.”

It has been argued that persistence alone is a major cause for concern.^26^ If decision makers would like to include persistence alone in MCDA-ACA, they would need to add P as a higher-level objective (see Supporting Information-2—MCDA-ACA + P). As a consequence, some additional hypothetical substances are classified as regrettable. Figure 4 shows the distribution of the MCDA outputs (*f*) of the hypothetical substance data set when the data set is assessed according to the three different objective hierarchies of MimicREACH, MCDA-ACA, and MCDA-ACA + P. As the distribution shifts from MimicREACH to MCDA-ACA and finally to MCDA-ACA + P, a greater share of the distribution (and therefore hazard combinations) lies below the categorization threshold of *f* = 0.17 and is therefore categorized as “regrettable.” Figure 4 also gives the example of two non-SVHC hazard combinations whose categorization changes from “not regrettable” to “regrettable” as the objective hierarchy expands to include more hazard combinations. The first hazard combination describes vBvT_eco_ substances that have low hazards in the other end points. The second one represents a vP substance with low hazards in the other end points.

In order to enable practitioners to use MCDA-ACA + P as well, it is also implemented in the Supporting Information-3 (under “MCDA-ACA + P - forUsers”).

## Discussion

### MCDA Method Parameters

MCDA is a method that has been recommended for use in REACH Authorisations and Restrictions.^27^ However, the precise method parameters most appropriate for the assessment of chemical alternatives have not been extensively discussed. In response to this need, we here investigate and recommend MCDA method parameters for the ACA, specifically objective hierarchies, aggregation, curvature of the value functions and weights, and the introduction of a classification threshold. Prior to this paper, to the best of our knowledge, all MCDA-MAVT parameters found in the literature for the ACA were the default method parameters of a simple objective hierarchy, equal weighting, additive aggregation, and linear value functions. In the context of the ACA, these parameters are not recommended, as the underlying decision logic cannot reproduce hazard assessment according to Article 57 of REACH. This is shown in London et al.^7^ For the exact alignment with Article 57 of REACH, we recommend using the parameters of MimicREACH; in a broader context, we propose to use MCDA-ACA as MCDA-ACA covers more objectives. Specifically, substances that are vPvT_eco_ and substances that are vBvT_eco_ are rated regrettable with MCDA-ACA, which is not the case for MimicREACH (and Article 57 of REACH). This means that the MCDA-ACA method is more stringent than Article 57 of REACH. Additionally, MCDA-ACA includes the higher-level objectives PMT_eco_, PM, ODP, and GWP, which are not included in MimicREACH.

The objectives currently defined in MCDA-ACA include, with one exception (the physical hazard of flammability), all minimum hazard criteria that were defined by the OECD in 2021.^18^ However, as MCDA-ACA is a flexible method, it can easily be adapted to include additional hazards if required. The flexibility of MCDA-ACA is also a strength in contrast to previously used methods that are more rigid such as set decision trees as used in GreenScreen.

In MCDA-ACA as well as in MimicREACH and MimicGreenScreen, the attributes used (e.g., the half-life in soil) are converted into discrete values (hazard levels of very high, high, moderate, and low). This conversion results in a loss of granularity, as there are no continuous values anymore but just four hazard levels. In our opinion, however, conversion into discrete values is important, if not unavoidable, for two reasons. First, different jurisdictions use different thresholds. Discrete values make it easier to adapt the method to jurisdictions outside the EU. For example, Article 57 of REACH states that a substance is bioaccumulative if the BCF is ≥2000.^22^ However, the BCF threshold for bioaccumulation in the US is ≥1000.^28^ The thresholds can be easily changed with discrete values, while it is more difficult to implement a new continuous value function where these thresholds are still met. Second, and maybe even more important, Article 57 of REACH is very specific on which hazard combinations are potential SVHCs (and therefore regrettable) and which ones are not. Even with the discrete values, it was only just possible (and with the help of the scaling factors) to correctly assign the hazard combinations to regrettable and nonregrettable. It would be very difficult to guarantee that all hazard combinations would always be correctly assigned when a continuous value function is used.

### Uncertainties in the Input Data and Missing Data

Uncertainties in the substance-specific data entering the method were not addressed in this work. One option for incorporating uncertainties would be to run the evaluation several times, each time varying one of the uncertain data points (e.g., the half-life of the chemical in water) while keeping all the other input data points constant. This can be carried out as a sensitivity analysis, where each of the uncertain data points is varied by the same percentage. Alternatively, the lowest and highest realistic values for any uncertain data point might be used (bounding analysis).^29^ This allows the user to estimate whether the data point within its bounds has a strong influence on the results or not. Future work could also incorporate the data uncertainties qualitatively. For example, it would be possible to evaluate the quality of the input data on a standardized scale (e.g., 1 could be “no information available,” 2 could be “data uncertain,” 3 “data certain,” and 4 could be “data very certain”). The uncertainty scores could then be aggregated for the lower-level objectives with a minimum aggregation, meaning that if, e.g., in PBT_eco_, P has an uncertainty score of 3, B a score of 1, and T_eco_ a score of 2, the overall score of PBT_eco_ would be 1.

Another critical point, as in all methods that evaluate chemical alternatives, is missing data on chemical attributes, i.e., hazardous properties. However, by using a minimum, rather than an additive aggregation, for the higher-level objectives, the availability of data for a higher-level objective that indicates that the alternative is a regrettable substitute is sufficient to classify this alternative. Missing data for other higher-level objectives do not influence this assessment. However, missing data do influence the assessment if the MCDA outcome is “not regrettable” because additional data on insufficiently characterized hazards may change the outcome to “regrettable.” We recommend in those cases using calculated and/or predicted attributes to provide the missing data points. When calculated or predicted attributes are used, a sensitivity analysis using these values is recommended. The Supporting Information-1 Section S6 gives guidance on which methods to use. Additional guidance is provided by the OECD.^18^

### Safe and Sustainable by Design

MCDA is a decision-making support tool that can assist in the design of safe and sustainable chemicals.^14,30^ Therefore, the MCDA-ACA method could be a relevant tool for stakeholders to achieve the objectives of the EU’s Green New deal. Dias et al.^31^ presented seven requisites underpinning an overall evaluation procedure for Safe and Sustainable by Design (see also Supporting Information-1 Section S5). MCDA-ACA fulfills most of these requisites. Specifically, the aggregation in MCDA-ACA does not allow for trade-offs between objectives, and the higher-level objectives are associated with regulatory reference points that act as classification criteria; thus, the assessment is absolute and not relative. MCDA-ACA currently does not take data quality into account, something that might need to be addressed in the future. Given the flexibility of MCDA-ACA, it is also possible to include more higher-level objectives including those that are suggested in Caldeira et al.,^32^ such as explosiveness or flammability.

### Implications

Methods for assessing alternatives to hazardous chemicals in a comprehensive way are much needed, and we hope that MCDA-ACA is a step forward in this direction. However, hazard assessment methods including MCDA-ACA should not be treated as black boxes. It is important to understand the way these methods work in order to communicate the results. Being transparent about how the results were obtained is especially important in larger companies, trade organizations, or governmental agencies.^6^ It might also be necessary to include other objectives such as costs or performance in the MCDA methods and to consider not only chemical-by-chemical substitution but also functional substitution or substitution by a change of the system.^1^

In any case, more should be done than just looking through certain lists (e.g., the Candidate List of SVHCs for Authorisation^33^) to decide whether a substance is a nonregrettable alternative. As pointed out by Slunge et al.,^2^ the European Commission^34^ estimated in 2006 that there were approximately 1500 substances with known SVHC properties and committed to “having all relevant currently known SVHCs included in the Candidate List by 2020“.^2,35^ However, by June 2024, only 240 substance entries were included in the Candidate List, including around 450 substances, which shows that those lists are not sufficient to identify regrettable substitutes.

## Supporting Information Available

Definitions for some of the terms used in the article, thresholds that we suggest to use with MCDA-ACA and MimicREACH, reasons for including scaling factors, deviation of the data classification threshold for MimicGreenScreen, and requisites laid down in Table 2 of Dias et al.^31^ (PDF)
Supporting Information-2 and -3: MCDA-ACA as well as MCDA - MimicREACH and MCDA-ACA + P for the use by practitioners (XLSX, XLSX)

## References

[ref1] Tickner J. A.; Schifano J. N.; Blake A.; Rudisill C.; Mulvihill M. J. Advancing safer alternatives through functional substitution. Environ. Sci. Technol. 2015, 49, 742–749. 10.1021/es503328m. 25517452

[ref2] Slunge D.; Andersson I.; Sterner T. REACH authorisation and the substitution of hazardous chemicals: The case of trichloroethylene. J. Clean. Prod. 2022, 364, 132637 10.1016/j.jclepro.2022.132637.

[ref3] Jacobs M. M.; Malloy T. F.; Tickner J. A.; Edwards S. Alternatives assessment frameworks: Research needs for the informed substitution of hazardous chemicals. Environ. Health Perspect. 2016, 124, 265–280. 10.1289/ehp.1409581. 26339778 PMC4786344

[ref4] Tickner J.; Jacobs M.; Malloy T.; Buck T.; Stone A.; Blake A.; Edwards S. Advancing alternatives assessment for safer chemical substitution: A research and practice agenda. Integr. Environ. Assess. Manag. 2019, 15, 855–866. 10.1002/ieam.4094. 30117284

[ref5] Bechu A. M.; Roy M. A.; Jacobs M.; Tickner J. A. Alternatives assessment: An analysis on progress and future needs for research and practice. Integr. Environ. Assess. Manag. 2024, 20 (5), 1337–1354. 10.1002/ieam.4882. 38124425

[ref6] Beaudrie C.; Corbett C. J.; Lewandowski T. A.; Malloy T.; Zhou X. Evaluating the Application of Decision Analysis Methods in Simulated Alternatives Assessment Case Studies: Potential Benefits and Challenges of Using MCDA. Integr. Environ. Assess. Manag. 2021, 17, 27–41. 10.1002/ieam.4316. 32681741

[ref7] London R.; Glüge J.; Scheringer M. Are Hazard Assessment Methods in the Assessment of Chemical Alternatives Suitable for REACH? Environ. Sci. Technol. 2024, 58, 10.1021/acs.est.4c03979. PMC11500412 39382051

[ref8] Belton V.; Stewart T. J. Analytical Biochemistry; Springer US: Boston, MA, 2002; Vol. 11, pp 1–5.

[ref9] Eisenführ F.; Weber M.; Langer T. Rational Decision Making; Springer Berlin Heidelberg: Berlin, Heidelberg, 2010.

[ref10] Zheng Z.; Arp H. P. H.; Peters G.; Andersson P. L. Combining In Silico Tools with Multicriteria Analysis for Alternatives Assessment of Hazardous Chemicals: Accounting for the Transformation Products of decaBDE and Its Alternatives. Environ. Sci. Technol. 2021, 55, 1088–1098. 10.1021/acs.est.0c02593. 33381962 PMC7871322

[ref11] Zheng Z.; Peters G. M.; Arp H. P. H.; Andersson P. L. Combining in Silico Tools with Multicriteria Analysis for Alternatives Assessment of Hazardous Chemicals: A Case Study of Decabromodiphenyl Ether Alternatives. Environ. Sci. Technol. 2019, 53, 6341–6351. 10.1021/acs.est.8b07163. 31081616

[ref12] Malloy T. F.; Sinsheimer P. J.; Blake A.; Linkov I. Use of multi-criteria decision analysis in regulatory alternatives analysis: A case study of lead free solder. Integr. Environ. Assess. Manag. 2013, 9, 652–664. 10.1002/ieam.1449. 23703936

[ref13] van Dijk J.; Figuière R.; Dekker S. C.; van Wezel A. P.; Cousins I. T. Managing PMT/vPvM substances in consumer products through the concepts of essential-use and functional substitution: a case-study for cosmetics. Environ. Sci. Process. Impacts 2023, 25 (6), 1067–1081. 10.1039/D3EM00025G. 37199459

[ref14] van Dijk J.; Flerlage H.; Beijer S.; Slootweg J. C.; van Wezel A. P. Safe and sustainable by design: A computer-based approach to redesign chemicals for reduced environmental hazards. Chemosphere 2022, 296, 134050 10.1016/j.chemosphere.2022.134050. 35189194

[ref15] Keeney R. L.; Raiffa H. Decisions with Multiple Objectives: Preferences and Value Trade-Offs; Cambridge University Press, 1993.

[ref16] US EPA Design for the Environment Alternatives Assessments. 2024; https://www.epa.gov/saferchoice/design-environment-alternatives-assessments.

[ref17] ECHA Online Training on Analysis of Alternatives. 2020; https://echa.europa.eu/online-training-on-analysis-of-alternatives.

[ref18] OECD Guidance on Key Considerations for the Identification and Selection of Safer Chemical Alternatives (Series on Risk Management No. 60); 2021; pp 1–55.

[ref19] Marttunen M.; Belton V.; Lienert J. Are objectives hierarchy related biases observed in practice? A meta-analysis of environmental and energy applications of Multi-Criteria Decision Analysis. Eur. J. Oper. Res. 2018, 265, 178–194. 10.1016/j.ejor.2017.02.038.

[ref20] Cinelli M.; Coles S. R.; Kirwan K. Analysis of the potentials of multi criteria decision analysis methods to conduct sustainability assessment. Ecol. Indicat. 2014, 46, 138–148. 10.1016/j.ecolind.2014.06.011.

[ref21] Belton V.. In In Multicriteria Decis. Mak. Adv. MCDM Model. Algorithms, Theory, Appl.; Gal T., Stewart T. J., Hanne T., Eds.; Springer US: Boston, MA, 1999; pp 335–366.

[ref22] European Chemicals Agency Guidance on Information Requirements and Chemical Safety Assessment Chapter R.11: PBT/vPvB Assessment - Version 4.0; 2023; pp 1–205.

[ref23] ECHA SVHC Support Document – HFPO-DA and its Salts/acyl Halides; 2019.

[ref36] Scheringer M.; Strempel S.; Hukari S.; Ng C. A.; Blepp M.; Hungerbühler K. How many persistent organic pollutants should we expect?. Atmos. Pollut. Res. 2012, 3, 383–391. 10.5094/APR.2012.044.

[ref24] EC Regulation (EC) No 1907/2006 of the European Parliament and of the Council Concerning the Registration, Evaluation, Authorisation and Restriction of Chemicals (REACH). 2006; http://data.europa.eu/eli/reg/2006/1907/oj/eng.

[ref25] GreenScreen Clean Production Action - GreenScreen® for Safer Chemicals® Method Documents. 2018; https://www.greenscreenchemicals.org/learn/guidance-and-method-documents-downloads.

[ref26] Cousins I. T.; Ng C.; Wang Z.; Scheringer M. Why is High Persistence Alone a Major Cause of Concern?. Environ. Sci. Process. Impacts 2019, 21, 781–792. 10.1039/C8EM00515J. 30973570

[ref27] ECHA Guidance on the Preparation of Socio-Economic Analysis as Part of an Application for Authorisation. 2011, 260.

[ref28] Gobas F. A. P. C.; de Wolf W.; Burkhard L. P.; Verbruggen E.; Plotzke K. Revisiting Bioaccumulation Criteria for POPs and PBT Assessments. Integr. Environ. Assess. Manag. 2009, 5, 624–637. 10.1897/IEAM_2008-089.1. 19552497

[ref29] Granger Morgan M. The neglected art of bounding analysis. Environ. Sci. Technol. 2001, 35 (7), 162 A–164 A. 10.1021/es012311v 11348103

[ref30] Caldeira F. R.; Garmendia Aquirre I.; Mancini L.; Tosches D.; Amelio A.; Rasmussen K.; Rauscher H.; Riego Sintes J.; Sala S. Safe and Sustainable by Design - Chemicals and Materials - Framework for the Definition of Criteria and Evaluation Procedure for Chemicals and Materials, 2022, pp 1–185.

[ref31] Dias L. C.; Caldeira C.; Sala S. Multiple criteria decision analysis to support the design of safe and sustainable chemicals and materials. Sci. Total Environ. 2024, 916, 169599 10.1016/j.scitotenv.2023.169599. 38151130

[ref32] Caldeira C.; Farcal R.; Moretti C.; Mancini L.; Rauscher H.; Rasmussen K.; Riego J.; Sala S. Safe and Sustainable by Design - Chemicals and Materials - Review of Safety and Sustainability Dimensions, Aspects, Methods, Indicators, and Tools; JRC Publications Repository, 2022.

[ref33] ECHA Candidate List of Substances of Very High Concern for Authorisation. 2024; https://echa.europa.eu/candidate-list-table.

[ref34] EC Q and A on the New Chemicals Policy; REACH. 2006; https://ec.europa.eu/commission/presscorner/detail/en/MEMO_06_488.

[ref35] EC Roadmap on Substances of Very High Concern. 2013; https://data.consilium.europa.eu/doc/document/ST-5867-2013-INIT/en/pdf.

