# Supplementary material for: Multiple-Criteria Decision Analysis for Assessments of Chemical Alternatives (MCDA-ACA)
Source: Environ Sci Technol. 2024 Oct 18;58(43):19315–24. doi: 10.1021/acs.est.4c03980 (PMC11526351; doi:10.1021/acs.est.4c03980)
Supplement: Supplementary file 1 — es4c03980_si_001.pdf [file es4c03980_si_001.pdf]

Supporting Information-1 to "Multi-Criteria Decision Analysis for Assessments of Chemical Alternatives (MCDA-ACA)"

Rachel London, Juliane Glüge,* and Martin Scheringer

Institute of Biogeochemistry and Pollutant Dynamics, ETH Zürich, 8092 Zürich, Switzerland

E-mail: juliane.gluege@usys.ethz.ch

Summary: 27 pages, 8 tables, 0 figures

Contents

S1 Definitions **S4**

S1.1 Objective S4

S1.2 Objective hierarchy, higher-level objectives, lower-level objectives S4

S1.3 Attributes S4

S1.4 Value Function S5

S1.5 Aggregation S5

S1.6 Weights S6

S2 Thresholds **S6**

S2.1 Persistence and bioaccumulation S7

S2.2 Ecotoxicity S8

S2.3 Human toxicity S9

S2.4 Mobility S9

S2.5 Ozone Depletion Potential S10

S2.6 Global Warming Potential S11

S3 Derivation of the MCDA parameters **S11**

S3.1 Thoughts and steps to derive the discrete values S11

S3.2 Reasons for including the scaling factors S12

S3.2.1 PBT_{eco} and PB S12

S3.2.2 PMT_{eco} and PM S13

S3.2.3 T_{hu} S13

S3.2.4 ODP and GWP S14

S4 Derivation of the data classification threshold for MimicGreenScreen **S15**

S5 Requisites laid out in Table 2 of Dias et al.[1] **S19**

S6 Possible *in-silico* tools to use **S20**

S6.1 Persistence S20

S6.2 Bioaccumulation S20

S6.3 Ecotoxicity S22

S6.4 Human toxicity S23

S6.5 Mobility S23

References **S24**

S1 Definitions

S1.1 Objective

The purpose of MCDA is to identify the best alternative in the context of specific criteria, preferences, and a given set of alternatives. For this, one must first establish objectives (i) against which the given alternatives can be evaluated. It should be noted that objectives are typically directional, i.e. one must define not only the desired quality but also the preferred direction, e.g. "low persistence" rather than just "persistence".[2] In this paper, however, the direction of the objectives has been omitted, i.e. 'persistence' rather than 'low persistence'. All objectives in this paper are hazards, so it can be assumed that all objectives have the same directionality - low hazard is always the desired direction of the objective.

S1.2 Objective hierarchy, higher-level objectives, lower-level objectives

An objective hierarchy shows visually how different objectives can be grouped together. Higher-level objectives can be broken down into constituent lower-level objectives.[2] For example, in this paper we identified the hazard combination PBT_{eco} as a higher-level objective composed of the three lower-level objectives of persistence (P), bioaccumulation (B), and ecotoxicity (T_{eco}).

S1.3 Attributes

Objectives are measured using attributes. An attribute is a metric by which the performance of an alternative can be determined. A single objective could have multiple possible attributes with different units.[2] For example, the objective of "persistence" could be measured by the attributes of "half-life in fresh water", or the "percentage dissolved organic carbon removed after 28 days" in the OECD 301 test. To transform the performance in

different attributes with different units into compatible values, a value function is required.[3]

S1.4 Value Function

Value functions are the mathematical expression of the decision maker's preferences, that are used to transform attributes of different units into compatible values. Selecting a curvature for the value function (e.g., linear, exponential, ect.) that correctly represents the decision maker's preferences is as an important part of the sensitivity analysis of an MCDA.[3] The transformed attributes can then be aggregated to generate a single value for each objective (ν_i, where $0.0 \leq \nu_i \leq 1.0$).[2] We recommend that the worst hazard level of all attributes is selected for the objective (thus to apply a minimum aggregation to the attributes).

For example, if there is a half-life for a certain substance in fresh-water, a half-life in marine water, a half-life in soil and a half-life in sediment then there needs to be a decision which hazard level persistence gets. It is proposed here that the worst outcome should be used to classify the objective (in this case persistence). Thus, if the half-life in soil gives a "very high" and all other attributes give only a "high" persistence, we propose to go forward with "very high". The reason is that it is not enough that the substance degrades in one compartment to be a non-regrettable alternative. It needs to degrade in all compartments. The same applies for ecotoxicity (it is not enough that the substance is not acute toxic, it also needs to be not chronic toxic), human toxicity (not only not carcinogenic but also not mutagenic and not toxic for reproduction) and partially also bioaccumulation. For bioaccumulation, there is just one threshold in Article 57 of REACH, but the guidance on PBT/vPvB assessment also refers to other endpoints/attributes and they – in a weight of evidence approach – could lead to worse results.

S1.5 Aggregation

To calculate an MCDA output for each alternative (f), the values for each of the objectives (ν_i) must be aggregated.[2] The aggregation equation selected should reflect the degree

of trade-off permissible between objectives, based on the decision maker's preferences.[4] For example, if poor performance in one objective should be entirely compensated for by good performance in another objective, a weighted average can be used (Eq. S1). However, the extent of compensation possible between two objectives is dependent upon their corresponding weights. If unequal weights are applied to each of the objectives, only partial compensation, rather than full compensation, is possible. On the other hand, if poor performance in one objective cannot be compensated for, a minimum aggregation should be used (Eq. S2).

$$f_{\text{add}} = \sum_{i=1}^{n} w_i \cdot \nu_i \tag{S1}$$

$$f_{\text{min}} = \min(\nu_i) \tag{S2}$$

S1.6 Weights

Some aggregation equations (e.g., Eq. S1), require a weight to be assigned to each objective (w_i, where $0.0 \leq w_i \leq 1.0$ and $\sum w_i = 1.0$). The weight indicates the importance of achieving a given objective,relative to the other objectives, based on the preferences of the decision maker.[2,4]

S2 Thresholds

Regulatory thresholds must be incorporated into MCDA, if it is to align with current regulation. This is done during the Data Normalisation step of MCDA-ACA. In this section, "very high", "high", "moderate", and "low" thresholds are proposed for attributes relevant to the lower-level objectives detailed in the main paper. These thresholds are based on EU regulation (Article 57 of REACH and the classification, labelling and packaging (CLP) regulation) and supplemented with guidance frameworks (GreenScreen®[5] and Cradle to Cradle®[6]).

S2.1 Persistence and bioaccumulation

The thresholds for "high" and "very high" in Table S1 and S2, correspond to those thresholds given in Article 57 of REACH.[7] The "moderate" and "low" thresholds, reflect values found in both the GreenScreen®[5] and Cradle to Cradle®[6] guidance.

Table S1: **Persistence thresholds:** Thresholds taken from Article 57 of the REACH regulation (Annex XIII, Chapter R.11: PBT/vPvB assessment - Table R.11-1).[7] Gray thresholds are those not stated in regulation, but derived from GreenScreen®[5] and Cradle to Cradle®[6] guidance

	**Persistance classification (days)**			
	**very high**	**high**	**moderate**	**low**
**Half-life (marine water)**	60	60	≥ 16	<16
**Half-life (fresh or estuarine water)**	60	40	≥ 16	<16
**Half-life (marine sediment)**	180	180	≥ 16	<16
**Half-life (fresh or estuarine water sediment)**	180	120	≥ 16	<16
**Half-life (soil)**	180	120	≥ 16	<16

Table S2: **Bioaccumulation thresholds:** Thresholds taken from Article 57 of the REACH regulation (Annex XIII, Chapter R.11: PBT/vPvB assessment - Table R.11-1).[7] Gray thresholds are those not stated in regulation, but derived from GreenScreen®[5] and Cradle to Cradle®[6] guidance

	**Bioaccumulation classification**			
	**very high**	**high**	**moderate**	**low**
**Bioconcentration factor (in aquatic species)**	≥ 5000	≥ 2000	≥ 500	< 500

S2.2 Ecotoxicity

The lower-level objective of low ecotoxicity can be determined by either the attribute of acute ecotoxicity or chronic toxicity, typically from algae, daphnia, or fish, with preference given to data from the most sensitive species. For acute toxicity, only a single threshold ("very toxic") is defined in CLP (Annex 1, Section 4.1, Table 4.1.0),[8] after which the thresholds were filled in ascending magnitudes of 10, as is done in the GreenScreen®[5] and Cradle to Cradle®[6] guidance. For chronic toxicity, there are several different thresholds listed as a what constitutes a "toxic" substance in different REACH and CLP regulatory guidance. For example, the no-observed effect concentration (NOEC) threshold for a substance to be considered toxic in REACH Article 57 is $\leq$ 0.01 mg/L, whereas in the CLP guidance it could be $\leq$ 0.01 mg/L, $\leq$ 0.1 mg/L, or $\leq$ 1.0 mg/L, depending on other physicochemical properties of the substance. The most conservative thresholds found are given in Table S3, but the user may want to review these to check they align with their situation. The "very toxic" threshold was set slightly below 0.01 mg/L.

Table S3: **Ecotoxicity thresholds:** Thresholds taken from CLP (Annex 1, section 4.1, table 4.1.0)[8] and Article 57 of the REACH regulation (Annex XIII, Chapter R.11: PBT/vPvB assessment - Table R.11-1).[7] Gray thresholds are those not stated in regulation, but suggested by the authors

	**Ecotoxicity classification (mg/L)**			
	**very high**	**high**	**moderate**	**low**
**Acute Toxicity - EC50 or LC50**[5,6,8]	≤ 1	≤ 10	≤ 100	$100 <$
**Chronic Toxicity - The long-term no-observed effect concentration (NOEC) or EC10**[8]	≤ 0.009	≤ 0.01	≤ 0.1	$0.1 <$

S2.3 Human toxicity

For Human Health Toxicity the attributes explicitly listed in Article 57 of REACH are shown in Table S4. There are additional thresholds and attributes suggested in other regulation (e.g. CLP[8]) and guidances (GreenScreen®[5] and Cradle to Cradle®[6]). However, we have only included the thresholds stated in Annex XIII of REACH for "high" human toxicity here and we would advice users of this method to check with the regulations in their jurisdiction for thresholds of "very high", "moderate" and "low". Important to note is here that "very high" and "high" human toxicity lead to the classification of the substances as regrettable substitute whereas moderate and low do not lead to this classification.

Table S4: **Human toxicity thresholds:** REACH regulation Annex XIII, Chapter R.11: PBT/vPvB assessment - Table R.11-1

	**Human toxicity classification**			
	**very high**	**high**	**moderate**	**low**
**Carcinogenic***		category 1A or 1B		
**Germ cell mutagenic***		category 1A or 1B		
**Toxic for reproduction***		category 1A, 1B, or 2		
**Evidence of chronic toxicity - specific target organ toxicity after repeated exposure (STOT RE2)**		category 1 or 2		

* according to CLP regulation (Regulation EC No 1272/2008)

S2.4 Mobility

According to Commission Delegated Regulation (EU) 2023/707 (ref[9]), a substance shall be considered 'to fulfil the mobility criterion (M) when the log K_{OC} is less than 3. For an ionisable substance, the mobility criterion shall be considered fulfilled when the lowest log K_{OC} value for pH between 4 and 9 is less than 3.' Commission Delegated Regulation (EU) 2023/707 also states 'A substance shall be considered to fulfil the 'very mobile' criterion

(vM) when the log K_{OC} is less than 2. For an ionisable substance, the mobility criterion shall be considered fulfilled when the lowest log K_{OC} value for pH between 4 and 9 is less than 2.'

Table S5: **Mobility thresholds:** Commission Delegated Regulation (EU) 2023/707 (ref[9])

	**Mobility classification**			
	**very high**	**high**	**moderate**	**low**
**log K_{OC}**	< 2	< 3	≥ 3	

S2.5 Ozone Depletion Potential

For Ozone Depleting Potential (ODP), the thresholds are derived from Annex I of Regulation (EC) No 1005/2009 on substances that deplete the ozone layer.[10] A threshold of 1.0, equivalent to the ODP of CFC-11 (a standard reference for ODP measurements and a common value in Annex I), is designated as very high. This benchmark aligns with recognized standards and reflects prevalent ODP values. The high ODP threshold is set at 0.1, as this is the lowest ODP value in Annex I's Groups I to VII. The medium threshold was set at 0.01, an order of magnitude below the "high" threshold. Substances with "very high" or "high" ODP are classified as regrettable substitutes whereas substances with low or medium ODP are not classified as regrettable substitutes.

Table S6: **Ozone Depleting Potential:** The thresholds for "very high" and "high" were derived from Annex I of Regulation (EC) No 1005/2009 on substances that deplete the ozone layer[10]

	**Classification**			
	**very high**	**high**	**moderate**	**low**
**Ozone Depleting Potential (ODP)**	≥ 1.0	≥ 0.1	≥ 0.01	< 0.01

S2.6 Global Warming Potential

In this study, the thresholds for categorizing Global Warming Potential (GWP) as ”very high”, ”high”, and ”medium” are established based on the precedent set in Annex IV of the proposed EU Regulation (EU) on fluorinated greenhouse gases.[11] Specifically, the lowest GWP values listed in Annex IV (150 and 10) are assigned as threshold to the levels ”very high” and ”high”. The medium threshold is 1.0, the value equal to that of CO_2. Substances with ”very high” or ”high” GWP are classified as regrettable substitutes whereas substances with low or medium GWP are not classified as regrettable substitutes.

Table S7: **Global Warming Potential (GWP):** based on values listed in Annex IV of the proposed EU Regulation (EU) on fluorinated greenhouse gases.[11] The values are used in the Annex IV for a different purpose and we adapted them here for the MCDA method.

	Classification			
	**very high**	**high**	**moderate**	**low**
**Global Warming Potential (GWP)**	≥ 150	≥ 10	≥ 1	< 1

S3 Derivation of the MCDA parameters

S3.1 Thoughts and steps to derive the discrete values

As described in the main article under “Parameters of the MCDA-ACA method”, it was clear that a convex value function is needed. However, the shape of the value function and then also the discrete values also had to be determined. We started with setting the value for “very high” to 0.1 and the value for “high” to 0.25. It then soon became apparent that the hazard combinations with high (high P, high B and high T_{eco} or just high T_{hu}) that need to be “regrettable” according to Article 57 of REACH will have higher scores than some hazard combinations that should not be regrettable such as high P and very high B. We included

the scaling factor, as described in the main article and saw that “high” has to be lower than 0.26. Using for example 0.3 for “high” would give the non-regrettable hazard combination of high P and very high B a score of 0.2 but also the regrettable hazard combination high P, high B and high T_{eco} a score of 0.2. We also found that the value for “moderate” has to be higher than 0.57. The combination of very high P, very high B and moderate T_{eco} needs to be (after including the scaling factor) above 0.17 as this hazard combination is according to Article 57 of REACH not regrettable. Using e.g., 0.56 for moderate would give this combination a score of 0.169 which would be “regrettable”. We choose at the end 0.1, 0.25, 0.6 and 1 for very high, high, moderate and low, respectively to have not so crooked numbers but still numbers that reflect a convex curvature. Using e.g., 0.1, 0.2, 0.6 and 1 would not give a convex value function, as the difference between high and moderate would be the same as between moderate and low.

S3.2 Reasons for including the scaling factors

MCDA-ACA, MimicREACH as well as MimicGreenScreen use for some higher-level objectives a scaling factor. The following sections lay down in detail, why this factor is needed for each of the respective higher-level objectives

S3.2.1 PBT_{eco} and PB

The lower-level objectives P and B occur in several of the higher-level objectives including in PBT_{eco} and PB. Article 57 of REACH states that substances that have high or very high persistence, high or very high bioaccumulation potential and high or very high ecotoxicity are potential SVHCs. Article 57 of REACH also states that substances that have very high persistence and very high bioaccumulation potential independent of the status of ecotoxicity are also potential SVHCs. However, this also implies that substances that have high persistence and a very high bioaccumulation potential (and low or medium ecotoxicity) are not potential SVHCs.

This means for the MCDA that in one case (PBT_{eco}) the average of high, high and high needs to be below the classification threshold of 0.17 and in the other case (PB) that the average of very high and high needs to be above the classification threshold of 0.17. As "very high" has in our case a lower value than "high", it is not possible to fulfill this requirement without a scaling factor. With a scaling factor of 2/3 and a value for "high" of 0.25, the score of PBT_{eco} is lowered by 2/3 which makes it possible to classify substances that have high persistence, high bioaccumulation potential and high ecotoxicity as potential SVHCs (score $\leq$ 0.17) and at the same time to classify substances that have high persistence and are very bioaccumualtive not as potential SVHCs (score $\geq$ 0.17).

S3.2.2 PMT_{eco} and PM

The reason for including the scaling factor in PMT_{eco} is very similar to the reason to include the scaling factor for PBT_{eco}. The idea is that substances with high or very high persistence, high or very high mobility and high or very high ecotoxicity should be classified as regrettable substitutes (in the case of MCDA-ACA) or as Benchmark 1 (in the case of MimicGreenScreen). Substances with high persistence and very high mobility (and low or medium ecotoxicity) as well as substances with very high persistence and high mobility (and low or medium ecotoxicity) should be non-regrettable substitutes (or Benchmarks 2 or higher). This is also here only possible with a scaling factor that lowers the score of PMT_{eco}.

S3.2.3 T_{hu}

The GreenScreen® decision tree suggests that substances that have very high human toxicity (Group I human) and high human toxicity (Group I human) should be classified as Benchmark 1. We have adopted this for MCDA-ACA and decided that substances that have high or very high human toxicity should be classified as regrettable substitutes in MCDA-ACA. In order to classify high human toxicity as regrettable or Benchmark 1 using the classification threshold of 0.17 and the value function that had been developed for the other higher-level

objectives (concave), it was necessary to lower the score of T_{hu}. Using again the scaling factor of 2/3, the value of 0.25 for "high" was lowered to 0.167 which makes it possible to also classify high human toxicity as regrettable or Benchmark 1.

S3.2.4 ODP and GWP

There are no strict thresholds available for GWP or ODP that would need to be fulfilled to classify a substance as regrettable or non-regrettable substitute. We decided therefore to classify substances as regrettable if they had a high or very high OPD or high or very high GWP. (For the thresholds of high and very high, see Sections S2.5 and S2.6, reflectively.) As described for T_{hu}, the scaling factor is needed to lower the value of "high" from 0.25 to 0.167 so that "high" can also be classified as regrettable (is below the classification threshold of 0.17).

S4 Derivation of the data classification threshold for MimicGreenScreen

Table S8: High-level objectives of the MimicGreenScreen method and some examples of the qualitative hazard descriptions, together with their hazard values. Hazard descriptors in bold are explicitly mentioned in Annex 3 of the GreenScreen® Guidance.[5] For hazard descriptors (and their values) in red, MimicGreenScreen and the Benchmark Score from GreenScreen® do not match. The conversion from the qualitative hazard descriptions into the hazard values was done using very high (v) = 0.1, high (h) = 0.25, moderate (m) = 0.6, and low (l) = 1.0. A factor of 2/3 was included in the hazard values of PBT$_{\text{eco}}$ and T$_{\text{hu}}$.

**High-level objectives**	**Qual. hazard description**	**Hazard value**	**Qual. hazard description**	**Hazard value**	**Qual. hazard description**	**Hazard value**	**Qual. hazard description**	**Hazard value**
	Benchmark 1		Benchmark 2		Benchmark 3		Benchmark 4	
PBT$_{\text{eco}}$	**hP, hB, vT**$_{\text{eco}}$	0.13	**mP, mB, mT**$_{\text{eco}}$	0.40	vP, mB, lT$_{\text{eco}}$	0.38	**lP, lB, lT**$_{\text{eco}}$, lT$_{\text{hu}-1}$	1.00
	vP, hB, vT$_{\text{eco}}$	0.13	vP, hB, hT$_{\text{eco}}$	0.13	hP, mB, lT$_{\text{eco}}$	0.411		
	hP, vB, vT$_{\text{eco}}$	0.13	vP, hB, mT$_{\text{eco}}$	0.21	mP, mB, lT$_{\text{eco}}$	0.49		
	vP, vB, vT$_{\text{eco}}$	0.09	vP, mB, hT$_{\text{eco}}$	0.21	mP, lB, lT$_{\text{eco}}$	0.58		
			vP, mB, mT$_{\text{eco}}$	0.29				
			hP, vB, hT$_{\text{eco}}$	0.13				
			hP, vB, mT$_{\text{eco}}$	0.21				
			hP, hB, mT$_{\text{eco}}$	0.24				
			hP, mB, hT$_{\text{eco}}$	0.24				
			hP, mB, mT$_{\text{eco}}$	0.32				

High-level objectives	Qual. hazard description	Hazard value	Qual. hazard description	Hazard value	Qual. hazard description	Hazard value	Qual. hazard description	Hazard value
	Benchmark 1		Benchmark 2		Benchmark 3		Benchmark 4	
			mP, vB, hT_{eco}	0.21				
			mP, vB, mT_{eco}	0.29				
			mP, hB, hT_{eco}	0.24				
			mP, hB, mT_{eco}	0.32				
			mP, mB, hT_{eco}	0.32				
			vP, mB, vT_{eco}	0.18				
			hP, mB, vT_{eco}	0.21				
			mP, vB, vT_{eco}	0.18				
			mP, hB, vT_{eco}	0.21				
			mP, mB, vT_{eco}	0.29				
			hP, hB, hT_{eco}	0.167				
			vP, vB, mT_{eco}	0.18				
			vP, vB, hT_{eco}	0.10				
T_{hu-1}	**h**$\mathbf{T}_{hu-1}$	0.167	**m**$\mathbf{T}_{hu-1}$	0.40			lT_{hu-1}	0.67
	vT_{hu-1}	0.07						

**High-level objectives**	**Qual. hazard description**	**Hazard value**	**Qual. hazard description**	**Hazard value**	**Qual. hazard description**	**Hazard value**	**Qual. hazard description**	**Hazard value**
	Benchmark 1		Benchmark 2		Benchmark 3		Benchmark 4	
PB	**vP, vB**	0.1	**hP, hB**	0.25	vP, mB	0.35	lP, lB	1.0
			vP, hB	0.175	mP, vB	0.35		
			hP, vB	0.175	hP, mB	0.425		
					mP, hB	0.425		
					mP, mB	0.6		
					mP, lB *	0.8		
PT_{eco}	**vP, vT_{eco}**	0.1	**hP, mT_{eco}**	0.425	mP, vT_{eco}*	0.35	lP, lT_{eco}	1.0
			vP, mT_{eco}	0.35	mP, hT_{eco}	0.425		
			hP, hT_{eco}	0.25	mP, mT_{eco}	0.6		
			vP, hT_{eco}	0.175	mP, lT_{eco}*	0.8		
			hP, vT_{eco}	0.175				
BT_{eco}	**vB, vT_{eco}**	0.1	**hB, mT_{eco}**	0.425	mB, vT_{eco}*	0.35	lB, lt_{eco}	1.0
			vB, mT_{eco}	0.35	mB, hT_{eco}	0.425		
			hB, hT_{eco}	0.25	mB, mT_{eco}	0.6		
			vB, hT_{eco}	0.175	mB, l_{eco}*	0.8		

**High-level objectives**	**Qual. hazard description**	**Hazard value**	**Qual. hazard description**	**Hazard value**	**Qual. hazard description**	**Hazard value**	**Qual. hazard description**	**Hazard value**
	Benchmark 1		Benchmark 2		Benchmark 3		Benchmark 4	
			hB, vT_{eco}	0.175				
T_{eco}			**vT_{eco}**	0.1	**hT_{eco}**	0.25		
					**mT_{eco}**	0.6		
not included					**mP or mB**	–		
					**hP or hB**	–		
					**vP or vB**	–		

* This combination of low-level objectives is covered by another high-level objective.

S5 Requisites laid out in Table 2 of Dias et al.[1]

The seven requisites are:

1. The evaluation procedure can be applied to new chemicals and materials or to existing ones.

2. The evaluation procedure shall take into account the lack of data and data uncertainty

3. The result of the evaluation can be expressed either as a class of Safe and Sustainable by Design (poor, good, very good) or with a numerical score derived from the combination of the individual scores in each aspect.

4. A criterion is defined as an aspect with an assessment method and a minimum or maximum threshold or target values, on which a decision may be based.

5. The ambition of the Safe and Sustainable by Design is to move from relative (safer and more sustainable) to absolute (safe and sustainable) improvements ensuring that chemicals and materials are produced and used without exceeding acceptable boundaries.

6. The chemical/material should be considered Safe and Sustainable by Design if passing the criteria defined for safety and environmental sustainability

7. The evaluation procedure is underpinned by a hierarchical principle (p.46). A 'step score' and an 'overall Safe and Sustainable by Design score' could be developed considering the combination of scores. If the minimum criteria for safety dimension are not met, then the chemical/ material cannot be considered as Safe and Sustainable by Design

S6 Possible *in-silico* tools to use

The following subsections list *in-silico* tools and equations that might be used to fill data gaps for organic substances.

S6.1 Persistence

There are several models available in scientific publications but also in open access applications that can predict the ready biodegradability of organic substances. Examples of those scientific publications are Cheng et al.[12], Mansouri et al.[13], Cao and Leung[14], Blay et al.[15], Zhan et al.[16], Lunghini et al.[17], Huang and Zhang[18] and Yin et al.[19]. Open access applications that include models for ready-biodegradability include BIOWIN in EPI Suite™ (ref.[20]), VEGA[21] and OPERA.[22]

Fewer models are available for the prediction of actual half-lives in soil, sediment or water. One reason for this is that it is more difficult to predict half-lives as there are fewer experimental data on half-lives than on ready-biodegradation tests. Nevertheless, models are included in EPI Suite™ (BIOWIN 3)[20] and OPERA[22] that are ready to use for organic chemicals.

S6.2 Bioaccumulation

There have also been several models published that can predict the bioconcentration factor (BCF) in aquatic species. And there is also good agreement between the calculated values of these models for substances with a log K_{OW} between 1 and 5 (ref.[23]). However, we have shown in Glüge et al.[23] that the bioaccumulation models from EPI Suite™, OASIS Catalogic, ACD/Labs and the models of Veith et al.[24],[25] should not be used for hydrophobic substances with a log $K_{OW} > 6$. Instead, we propose in Glüge et al.[23] an alternative model for the BCF that is based on the freely dissolved chemical concentration in water and includes biotransformation of the compounds in the body. Basing the model on the freely dissolved

concentration in water is important as also fish can only take up the fraction that is freely dissolved. The following equations describe the basic set up of the model. More detailed information is provided in the SI-1 of Glüge et al.[23] As usual, the BCF is defined as:

$$\mathrm{BCF} = \frac{k_\mathrm{R}}{k_\mathrm{V} + k_\mathrm{E} + k_\mathrm{M} + k_\mathrm{G}} \tag{S3}$$

where the k-values are the rate constants for: k_R for respiratory uptake, k_V for respiratory loss, k_E for egestion, k_M for biotransformation, and k_G for growth dilution. These can be calculated from:

$$k_\mathrm{R} = \frac{1}{0.01 + \frac{1}{K_\mathrm{OW}}} W^{0.4} \tag{S4}$$

which yields k_R in units of [L kg^{-1} d^{-1}]. The fish weight, W, has to be in kg. K_OW is the octanol-water partition coefficient.

$$k_\mathrm{V} = k_\mathrm{R}/(L_\mathrm{B} \cdot K_\mathrm{OW}) \tag{S5}$$

where L_B is the lipid content of the organism.

$$k_\mathrm{E} = 0.125 \cdot k_\mathrm{D} \tag{S6}$$

where k_D is the rate constant for dietary update, defined as:

$$k_\mathrm{D} = 0.02 \cdot W^{-0.15} \cdot \frac{e^{0.006 \cdot T}}{5.1 \cdot 10^{-8} \cdot K_\mathrm{OW} + 2} \tag{S7}$$

T is here the temperature in °C

$$k_\mathrm{G} = 0.0005 \cdot W^{-0.2} \tag{S8}$$

In Glüge et al.[23], the following parameters were used: a fish weight of 10 g, a lipid content of the organism of 0.06 and a temperature of 20 °C. Values for the K_{OW} were obtained from COSMO*therm*[26], but values from OPERA[22] or reliable experimental values are also fine.

The biotransformation rate constant was calculated as the average of the values from six models: the three QSARIN models[27], the IFS model[28], the OPERA kM model[22] and the EPI Suite™ kM model[29,30]. The easiest way to obtain these values is (to the best of our knowledge) through EAS-E Suite.[31]

S6.3 Ecotoxicity

Different ecotoxicity models are included e.g., in the Danish (Q)SAR Database.[32] These models include acute aquatic toxicity models for fish, daphnid, and green algae. However, we could not find documentation of the models, so it is unclear on what data these models are based on. We used therefore in our previous publication, Rudin et al.[33], baseline toxicity models to calculate short-term and long-term toxicity to fish and aquatic invertebrates. The baseline toxicity is the minimal toxicity that a chemical can exhibit[34] The applied models were based on an extensive literature review that is provided in the SI-3 of Rudin et al.[33] Listed here are only the equations that were also selected in Rudin et al.[33], but other equations are available in the SI-3 of Rudin et al.[33]

The selected equation for the **short-term toxicity to fish** is based on LC_{50} values for 2-3 month old guppies (*Poecilia reticulata*) and was validated for organic neutral substances with a log $K_{OW} < 6$ (ref.[35]):

$$\log 1/\mathrm{LC}_{50}\ (\mathrm{L/mol}) = 0.907 \log K_{\mathrm{OW}} - 4.94 \tag{S9}$$

The selected equation for the **long-term toxicity to fish** is based on the NOEC after 28 to 32 days for effects in the early life stage of *Brachydanio rerio* (ref.[36]). The data were

validated for organic neutral substances with $1 < \log K_{\mathrm{OW}} < 6$:

$$\log \text{NOEC (mmol/L)} = -0.90 \log K_{\mathrm{OW}} + 0.7 \tag{S10}$$

The selected equation for the **short-term toxicity to aquatic invertebrates** is based on the immobilization of *Daphnia magna* after 48 hours (ref.[36]). The data were validated for organic neutral substances with $1 < \log K_{\mathrm{OW}} < 6$:

$$\log \text{EC}_{50} \text{ (mmol/L)} = -0.95 \log K_{\mathrm{OW}} + 1.68 \tag{S11}$$

The selected equation for the **long-term toxicity to aquatic invertebrates** is based on growth reproduction of *Daphnia magna* after 16 days and was validated for chemicals with $1 < \log K_{\mathrm{OW}} < 6$ (ref.[36]) :

$$\log \text{NOEC (mmol/L)} = -1.05 \log K_{\mathrm{OW}} + 1.15 \tag{S12}$$

S6.4 Human toxicity

Human toxicity models for different endpoints including estrogen receptor binding/inhibition, androgen receptor binding/inhibition, thyroid-related endpoints, carcinogenicity and others are included e.g., in the Danish (Q)SAR Database.[32]

S6.5 Mobility

The mobility of a substances is determined by the soil adsorption coefficient (log K_{OC}). If experimental log K_{OC} values are not available, COSMO*therm*[26] might be used to calculate the values for neutral substances. Otherwise, OPERA[22] or KOCWIN in EPI Suite™ are also able to calculate log K_{OC} values.

References

(1) Dias, L. C.; Caldeira, C.; Sala, S. Multiple criteria decision analysis to support the design of safe and sustainable chemicals and materials. *Science of The Total Environment* **2024**, *916*, 169599.

(2) Eisenführ, F.; Weber, M.; Langer, T. *Ration. Decis. Mak.*; Springer Berlin Heidelberg: Berlin, Heidelberg, 2010.

(3) Keeney, R. L.; Raiffa, H. *Decisions with Multiple Objectives: Preferences and Value Trade-Offs*; Cambridge University Press, 1993.

(4) Langhans, S. D.; Reichert, P.; Schuwirth, N. The method matters: A guide for indicator aggregation in ecological assessments. *Ecol. Indic.* **2014**, *45*, 494–507.

(5) GreenScreen Clean Production Action - GreenScreen® for Safer Chemicals® Method Documents. 2018; https://www.greenscreenchemicals.org/learn/guidance-and-method-documents-downloads.

(6) Cradle to Cradle Products Innovation Institute *Material health assessment methodology*; 2022; pp 1–96.

(7) European Chemicals Agency *Guidance on Information Requirements and Chemical Safety Assessment Chapter R.11: PBT/vPvB assessment - version 4.0*; 2023; pp 1–205.

(8) ECHA *Guidance on the Application of the CLP Criteria*; 2024; pp 1–644.

(9) EC *Commission Delegated Regulation (EU) 2023/707 of 19 December 2022 amending Regulation (EC) No 1272/2008 as regards hazard classes and criteria for the classification, labelling and packaging of substances and mixtures*; 2022.

(10) European Parliament and Council Regulation (EC) No 1005/2009 of 16 September 2009 on substances that deplete the ozone layer. 2009.

(11) European Commission Proposal for a regulation of the European Parliament and of the Council on fluorinated greenhouse gases, amending Directive (EU) 2019/1937 and repealing Regulation (EU) No 517/2014. 2022.

(12) Cheng, F.; Ikenaga, Y.; Zhou, Y.; Yu, Y.; Li, W.; Shen, J.; Du, Z.; Chen, L.; Xu, C.; Liu, G.; Lee, P. W.; Tang, Y. In silico assessment of chemical biodegradability. *J. Chem. Inf. Model.* **2012**, *52*, 655–669.

(13) Mansouri, K.; Ringsted, T.; Ballabio, D.; Todeschini, R.; Consonni, V. Quantitative structure-activity relationship models for ready biodegradability of chemicals. *J. Chem. Inf. Model.* **2013**, *53*, 867–878.

(14) Cao, Q.; Leung, K. M. Prediction of chemical biodegradability using support vector classifier optimized with differential evolution. *J. Chem. Inf. Model.* **2014**, *54*, 2515–2523.

(15) Blay, V.; Gullón-Soleto, J.; Gálvez-Llompart, M.; Gálvez, J.; García-Domenech, R. Biodegradability Prediction of Fragrant Molecules by Molecular Topology. *ACS Sustain. Chem. Eng.* **2016**, *4*, 4224–4231.

(16) Zhan, Z.; Li, L.; Tian, S.; Zhen, X.; Li, Y. Prediction of chemical biodegradability using computational methods. *Mol. Simul.* **2017**, *43*, 1277–1290.

(17) Lunghini, F.; Marcou, G.; Gantzer, P.; Azam, P.; Horvath, D.; Van Miert, E.; Varnek, A. Modelling of ready biodegradability based on combined public and industrial data sources. *SAR and QSAR in Environmental Research* **2020**, *31*, 171–186.

(18) Huang, K.; Zhang, H. Classification and Regression Machine Learning Models for Predicting Aerobic Ready and Inherent Biodegradation of Organic Chemicals in Water. *Environ. Sci. Technol.* **2022**, *56*, 12755–12764.

(19) Yin, H.; Lin, C.; Tian, Y.; Yan, A. Prediction and Structure-Activity Relationship Analysis on Ready Biodegradability of Chemical Using Machine Learning Method. *Chem. Res. Toxicol.* **2023**, *36*, 617–629.

(20) EPA, U. Estimation Programs Interface Suite™ for Microsoft® Windows, v 4.11. United States Environmental Protection Agency, Washington, DC, USA, 2012.

(21) Lombardo, A.; Pizzo, F.; Benfenati, E.; Manganaro, A.; Ferrari, T. *QMRF for VEGA Ready Biodegradation model*; 2022.

(22) Mansouri, K.; Grulke, C. M.; Judson, R. S.; Williams, A. J. OPERA models for predicting physicochemical properties and environmental fate endpoints. *Journal of cheminformatics* **2018**, *10*, 1–19.

(23) Glüge, J.; Escher, B. I.; Scheringer, M. How error-prone bioaccumulation experiments affect the risk assessment of hydrophobic chemicals and what could be improved. *Integr. Environ. Assess. Manag.* **2023**, *19*, 792–803.

(24) Veith, G. D.; DeFoe, D. L.; Bergstedt, B. V. Measuring and estimating the bioconcentration factor of chemicals in fish. *J. Fish. Res. Board Canada* **1979**, *36*, 1040–1048.

(25) Veith, G. D.; Macek, K.; Petrocelli, S.; Carroll, J. *Aquat. Toxicol.*; ASTM International, 1980; pp 116–14.

(26) Biovia COSMOtherm. 2020; `https://www.3ds.com/products-services/biovia/products/molecular-modeling-simulation/solvation-chemistry/biovia-cosmotherm/`.

(27) Papa, E.; van der Wal, L.; Arnot, J. A.; Gramatica, P. Metabolic biotransformation half-lives in fish: QSAR modeling and consensus analysis. *Sci. Total Environ.* **2014**, *470-471*, 1040–1046.

(28) Brown, T. N.; Arnot, J. A.; Wania, F. Iterative fragment selection: A group contribution approach to predicting fish biotransformation half-lives. *Environ. Sci. Technol.* **2012**, *46*, 8253–8260.

(29) Arnot, J. A.; Mackay, D.; Parkerton, T. F.; Bonnell, M. A database of fish biotransformation rates for organic chemicals. *Environ. Toxicol. Chem.* **2008**, *27*, 2263–2270.

(30) Arnot, J. A.; Mackay, D.; Bonnell, M. Estimating metabolic biotransformation rates in fish from laboratory data. *Environ. Toxicol. Chem.* **2008**, *27*, 341–351.

(31) ARC *EAS-E Suite (Ver.0.95-BETA, release Feb., 2022)*; 2022.

(32) DTU Danish (Q)SAR Models. 2024; `https://qsarmodels.food.dtu.dk/runmodel/index.html`.

(33) Rudin, E.; Glüge, J.; Scheringer, M. Per- and polyfluoroalkyl substances (PFASs) registered under REACH—What can we learn from the submitted data and how important will mobility be in PFASs hazard assessment? *Sci. Total Environ.* **2023**, *877*, 162618.

(34) Escher, B. I.; Hermens, J. L. Modes of action in ecotoxicology: Their role in body burdens, species sensitivity, QSARs, and mixture effects. *Environ. Sci. Technol.* **2002**, *36*, 4201–4217.

(35) Könemann, H. Quantitative structure-activity relationships in fish toxicity studies Part 1: Relationship for 50 industrial pollutants. *Toxicology* **1981**, *19*, 209–221.

(36) EC *Technical guidance document in support of Commission Directive 93/67/EEC on risk assessment for new notified substances and Commission Regulation (EC) No 1488/94 on risk assessment for existing substances: Part III*; 1996.
